# Study Design and Organization of the JPHC Study

**DOI:** 10.2188/jea.11.6sup_3

**Published:** 2007-11-30

**Authors:** Shaw Watanabe, Shoichiro Tsugane, Tomotaka Sobue, Masamitsu Konishi, Shunroku Baba

**Affiliations:** 1 National Cancer Center Research Institute.; 2 National Cardiovascular Center.

**Keywords:** cancer, cardiovascular disease, cohort study, epidemiology, prospective study, risk factors

## Abstract

The Japan Public Health Center-based Prospective Study on Cancer and Cardiovascular Diseases (JPHC Study; formerly called “Koseisho Multipurpose Prospective Cohort Study”) began in 1990(Cohort I) and 1993(Cohort II). The JPHC Study covers 11 public health center areas throughout Japan and includes a total of 140,420 residents. The study’s design includes a baseline survey with a self-administered questionnaire on lifestyle and collection of blood and health checkup data; a follow-up system for mortality, migration, and incidence of cancer and cardiovascular diseases; an additional follow-up survey after 5 and 10 years; and distribution of a newsletter. The JPHC study is expected to provide evidence for the prevention and control of cancer and cardiovascular diseases in the 21st century.

## INTRODUCTION

A large-scale population-based cohort study is a desirable means of elucidating risk factors for cancer and cardiovascular diseases. Often, it takes more than 10 years for these chronic degenerative diseases to become clinically detectable. Beginning in 1965, Soda, Hirayama and coworkers^1^^)^ followed a cohort of 265,000 subjects in 29 public health center areas of six prefectures in Japan. They found significant relationships between smoking and cancer and other diseases and a significant preventive effect of consuming green and yellow vegetables on cancer mortality. This cohort study had several methodological advantages, such as high response rate (approximately 95%) and low migration rate (8% for 17 years), however, the simplicity of the questionnaire caused several major limitations: participants were surveyed regarding only seven food items, green tea temperature preference, and smoking and alcohol use habits. Moreover, there have been drastic changes in lifestyle among the Japanese population during these decades, which makes lifestyle observed at baseline in this original cohort difficult to apply to the current Japanese population. The necessity of a new population-based prospective study was thus recognized and the Japan Public Health Center-based Prospective Study on Cancer and Cardiovascular Diseases (JPHC Study) was launched.

The study aims to clarify risk and preventive factors for cancer and cardiovascular diseases by a large-scale population-based prospective study. Furthermore, the obtained results will be useful for the future promotion of good health in Japan.

## METHODS

### Study cohorts

Between 1989 and 1991, the Epidemiology Division of the National Cancer Center Research Institute conducted a cross-sectional study, randomly selecting men aged 40 to 59 years and their wives from the general population in the territories of five public health centers in Ninohe (Iwate prefecture), Yokote (Akita prefecture), Saku (Nagano prefecture), Ishikawa (Okinawa prefecture), and Katsushika-kita (Tokyo metropolis). Blood and urine samples were collected and the applicability of biological markers to epidemiological research was heavily investigated^2^^, ^^3^^)^. Based on the experience of this cross-sectional study and with the cooperation of each public health center, cohorts consisting of 40- to 59-year-old residents were established in each public health center area at the end of 1989, and the baseline survey and follow-up were started in 1990. This group became known as cohort I. The primary purpose was to identify cancer risk factors with a protocol that incorporated health screening data and blood storage for future study.

Cohort II was added to the study in 1992, and data collection started in 1993. Similar data was collected from this cohort, but expanded to detect risk factors of both cancer and cardiovascular diseases. Six public health centers in Kashiwazaki (Niigata prefecture), Kasama (Ibaraki prefecture), Tosayamada (Kochi prefecture), Arikawa (Nagasaki prefecture), Miyako (Okinawa prefecture), and Suita (Osaka prefecture) joined the study.

### Organization

A study protocol was developed by the research members of the JPHC Study and authorized by the Ministry of Health and Welfare. The organization of the JPHC Study Group is listed in Table 1. Due to the different starting dates, the operating committees for cohort I and cohort II were separately organized, but they communicate closely with each other. A Steering Board Committee was instituted to manage and control the progress of the whole study, and an Advisory Board Committee and consultants helped the progress. To use the health checkup data from each study area, standardization of laboratory data was planned by the Standardization Committee. A Nutritional Study Committee was set up to focus on dietary assessment of the participants and evaluate the validity of the assessment method. Annual meetings with relevant offices in the Ministry of Health and Welfare are also held.

**Table 1.** Organization of JPHC study group (April 1, 1989 to March 31, 1997).

**Principal Investigator:** Keiichi Suemasu (National Cancer Center, 1989-1991), Tadao Kakizoe (National Cancer Center, 1991-1992), Shaw Watanabe (National Cancer Center; 1992-1997)

**Advisory Board Committee:** Shaw Watanabe (chief), Masamitsu Konishi (National Cardiovascular Center), Shigeaki Baba (Hyogo Medical Center for Adults), Suketami Tominaga (Aichi Cancer Center)

**Steering Board Committee:** Shaw Watanabe (chief), Shoichiro Tsugane (National Cancer Center), Tomotaka Sobue (National Cancer Center), Masamitsu Konishi, Shunroku Baba, Atsushi Terao (National Cardiovascular Center)

**Ethical Committee:** Hiroshi Inaba (consultant; Juntendo University)

**Cohort I Operation Committee:** Shoichiro Tsugane (chief), Shunroku Baba, Keigo Miyagawa, Fumihiko Saito (Ninohe Public Health Center), Yoshimichi Miyajima, Noriyuki Suzuki (Yokote Public Health Center), Hideki Sanada, Yoshiyuki Hatakeyama, Fumimune Kobayashi, Yuji Shirai (Saku Public Health Center), Yukimasa Kishimoto, Eikichi Takara, Masako Kinjo (Ishikawa Public Health Center), Kouhei Imoto, Heihachiro Yazawa, Takehisa Seo (Katsushika Public Health Center)

**Cohort II Operation Committee:** Tomotaka Sobue (chief), Atsushi Terao, Akira Murata, Koji Minato, Kazuo Motegi (Kasama Public Health Center), Kazumitsu Matsui (Kashiwazaki Public Health Center), Mitsunori Doi, Yoshinori Ishikawa, Atushi Terao (Tosayamada Public Health Center), Hiraku Sueta, Hiroshi Doi (Arikawa Public Health Center), Hachiro Sakiyama, Naokiyo Onga (Miyako Public Health Center), Fujiko Horii, Gosaburo Asano, Hidemi Yamaguchi (Suita Public Health Center)

**Standardization Committee:** Minoru Iida (chief; Osaka Medical Center for Cancer and Cardiovascular Diseases), Shosui Matsushima (Saku General Hospital)

**Nutritional Committee:** Masayuki Akabane (chief; Tokyo University of Agriculture), Momoko Yamaguchi (National Institute of Health and Nutrition), Shoichiro Tsugane, Atsushi Terao

**Consultants:** Presidents of the National Cancer Center and National Cardiovascular Center, Director of the Ministry of Health and Welfare, supportive members in the Ministry of Health and Welfare.

### Study subjects

**Figure 1.**
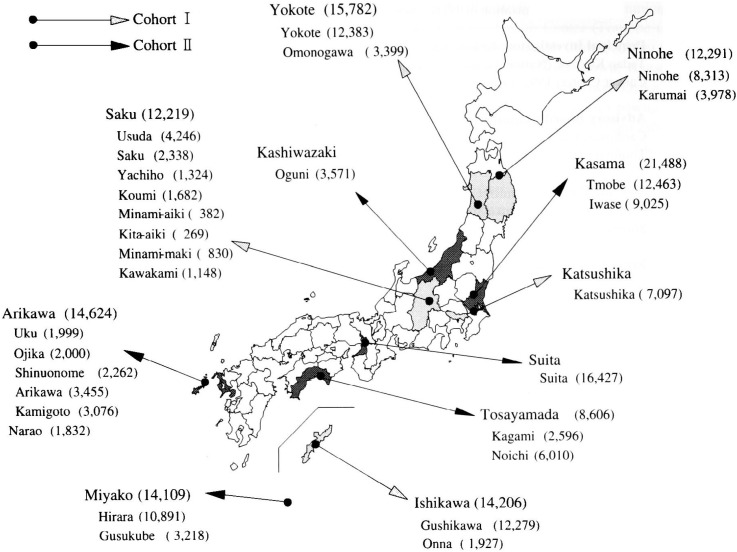
Location of study areas and number of study subjects.

Basically, all the residents within the age range 40-59 years, or 40-69 for cohort II, living in the city and town in each public health center area were selected as candidates. The location of the study areas and number of study subjects are shown in Figure 1. A total of 140,409 subjects were included based on municipality resident registry of 29 districts in the territories of 11 public health centers.

*Cohort I*: All residents, aged between 40 and 59 years on December 31, 1989, in the following areas were registered as the subjects for the study: Ninohe Public Health Center (Ninohe city, Karumai town), Yokote Public Health Center (Yokote city, Omonogawa town), Saku Public Health Center (Usuda town, Saku town, Koumi town, Kawakami village, Minamimaki village, Minamiaiki village, Kitaaiki village, Yachiho village), and Ishikawa Public Health Center (Gushikawa city, Onna village). Residents in the Katsushika Public Health Center area and Katsushika-kita Public Health Center area in Tokyo were called to join this study at the time of their age 40 or 50 health checkups, which are provided free to all the residents in Katsushika ward.

*Cohort II*: All residents aged between 40 and 69 years on December 31, 1992, in the following areas were registered as subjects for the study at the local municipality government: Kasama Public Health Center (Tomobe town, Iwase town), Kashiwazaki Public Health Center (Oguni town), Tosayamada Public Health Center (Noichi town, Kagami town), Arikawa Public Health Center (Uku town, Ojika town, Sinuonome town, Arikawa town, Kamigoto town, Narao town), and Miyako Public Health Center (Hirara city, Gusukube town). All residents aged 40 or 50 years in fiscal year 1993 in the Suita Public Health Center area in Osaka were selected. Another subcohort in Suita city was added. It was set up by the National Cardiovascular Center from 1989 to 1992 by random selection from the residents in Suita city aged 30 to 79 years, stratified by each 10-year age-sex group based on municipality population registry. Participants who still lived in Suita city on April 1, 1993, and were aged 40 to 69 years at that time were transferred to this study.

### Baseline survey

The details of baseline survey are described in another report^4^^)^. Briefly, a self-administered lifestyle questionnaire was provided to all cohort subjects. In 1990 the questionnaire was distributed mostly by hand or partly by mail in four populations in cohort I and in 1993 in five populations in cohort II. Incomplete answers were supplemented by telephone interviews. In the Katsushika Public Health Center area, questionnaires were administered on the occasion of health checkups from 1990 to 1994. In the Suita Public Health Center area, questionnaires were mailed to all the subjects from 1993 to 1995 and supplemented by interviews at participants’ health checkups or by telephone interviews for those who did not attend their health checkups.

A total of 10 ml blood was provided voluntarily by cohort subjects during their health checkups. The plasma and buffy layer were divided into four 1.0-ml tubes (three for plasma and one for the buffy layer) and stored at -80°C. The blood was collected from 1990 to 1992 (1990 to 1994 for the Katsushika Public Health Center cohort) in cohort I and from 1993 to 1995 in cohort II. The data from the participants’ health checkups were also obtained at this time.

### Follow-up system

The study subjects will be followed up for mortality, migration, and incidence of cancer and cardiovascular diseases such as stroke, myocardial infarction, and sudden death of unknown cause.

*Mortality:* Each public health center collects the death certificates of the study subjects and the information is sent to the coordinating centers annually in the format of a list of deaths. For outmigrated subjects, a coordinating center refers their vital status to the local government office of the new address and identifies the cause of death by linking with the death certificates collected centrally in the Information Bureau of the Ministry of Health and Welfare.

*Migration:* Each public health center reports every migration from an original address to a coordinating center by referring to each local government annually with information of new addresses. The coordinating center refers to those new addresses every four years thereafter.

*Incidence of cancer and cardiovascular diseases:* To collect incidence data of cancer and cardiovascular disease, two data sources are used: one from the local major hospital and the other from a population-based registry (usually prefecturewide). In some areas, major hospitals care for most cancer or cardiovascular patients (up to 80%). These hospitals are listed on death certificates. Then local major hospitals are considered as the primary data source, and clinical information is extracted from medical records into cohort-specific registration forms either by physicians in the hospital or physicians in the public health center. In this case, the population-based registry is used as a supplemental data source, if available. In those areas where local hospitals cannot cover a sufficiently high proportion of cancer or cardiovascular patients, data from local hospitals and population-based registry are considered equally as primary data sources. In this case, if the quality of the population-based registry is not high, additional activities are encouraged to improve the quality within the cohort subjects. Quality of registration within the cohort is monitored in all study areas using several indices: O/E ratio (observed versus expected ratio), I/D ratio (incidence versus mortality ratio). DCN% (proportion of death certificates notified), DCO% (proportion of death certificate only), and HV% (proportion of histologically verified cases).

### Follow-up surveys

**Figure 2.**
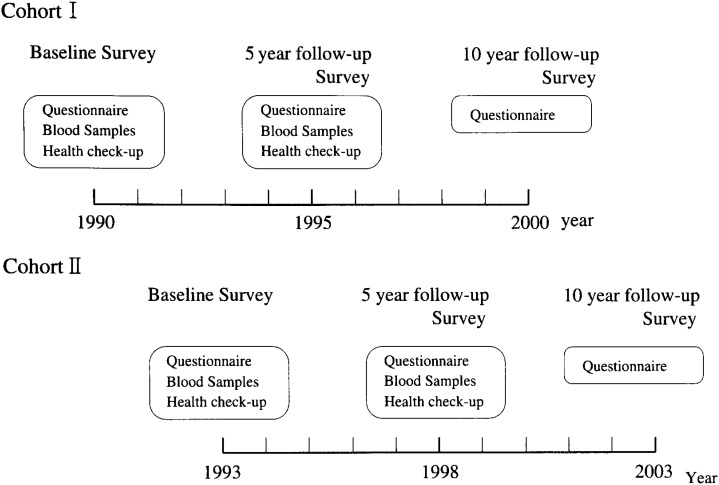
Research schedule of the JHPC Study.

A follow-up survey is scheduled 5 years after the baseline survey, which will include the questionnaire and the collection of blood and health checkup data. The purpose of this study is to assess dietary habits semi-quantitatively, changes of life habits, and disease history during 5 years. Another follow-up survey is also scheduled 10 years after the baseline survey, which will include only a questionnaire. Research scheduled at the 10-year follow-up survey is shown in Figure 2.

### Newsletter

Regular distribution of a newsletter to the study subjects is planned to inform them of the progress of the JPHC study and to provide health education. The coordinating center will prepare the central version that includes general findings and news. Each public health center will also prepare a local version. The time schedule of newsletter distribution will depend on the progress of the study.

## Discussion

After the completion of the cohort study by Hirayama et al.^1^^)^, several large-scale cohort studies were launched not only in Japan, but also in other countries. Among them, the JPHC study has several unique properties. First, public health centers were chosen to be the contact at the local level for coordinating all relevant facilities, such as local government, hospitals and health checkup providers, in order to collect information systematically. Because public health centers are primarily administrative branches of the prefectural government, they function well for this purpose. Also, it is relatively easy to standardize study methods throughout the entire study group.

Second, study subjects consist of all residents in defined areas, as identified from population registers. This makes it easy to generalize the findings obtained from the study. In addition, it is relatively easy to collect the data on mortality and migration because most information is available from the local government.

Third, study areas are distributed throughout Japan from north to south. This will maintain a sufficient magnitude of variation in lifestyle within the cohort, which makes internal comparison more powerful to detect significant associations.

Fourth, blood samples were collected and stored for the future use. This is particularly important because bacterial or viral infection, such as *helicobacter pylori*, hepatitis C virus, human papillomavirus, and human T-cell leukemia/lymphoma virus I are major risk factors for cancer. Also, biochemical substances in blood, such as micronutrients or carcinogenic compounds, may provide objective and quantitative information for an individual’s exposure to these chemicals. Moreover, critically important exposure information of carcinogenic chemicals or oxidative stress, namely level of DNA adducts, may be available using blood cell components. Further information on genetic polymorphism of metabolizing or repair enzyme using blood cell components will contribute to the investigation on gene-environment interactions.

Fifth, vital status is followed not only for those who remain living in the study area, but also for those who outmigrated from the study area, by individually asking local governments where the subjects have migrated to. This is especially important where migration rate is high.

Sixth, a questionnaire will be distributed at least three times for the fixed cohort. This will allow us to collect data on changing lifestyle and disease occurrence between the surveys.

Finally, a newsletter will be distributed to all study subjects during the follow-up period to inform them of the progress of the study. This will be important to keep the study subjects feeling actively involved in the study. Also, an outline of the JPHC Study has been posted on a website (http://www.east.ncc.go.jp/epi/jphc).

These unique features enable us to gather high-quality evidence, which will be essential to promote cancer and cardiovascular prevention strategies in Japan.
